# Supplementary material for: Comparative analysis of 1152 African-American and European-American men with prostate cancer identifies distinct genomic and immunological differences
Source: Commun Biol. 2021 Jun 3;4:670. doi: 10.1038/s42003-021-02140-y (PMC8175556; doi:10.1038/s42003-021-02140-y)
Supplement: Supplementary file 2 — Supplemental Information [file 42003_2021_2140_MOESM2_ESM.pdf]

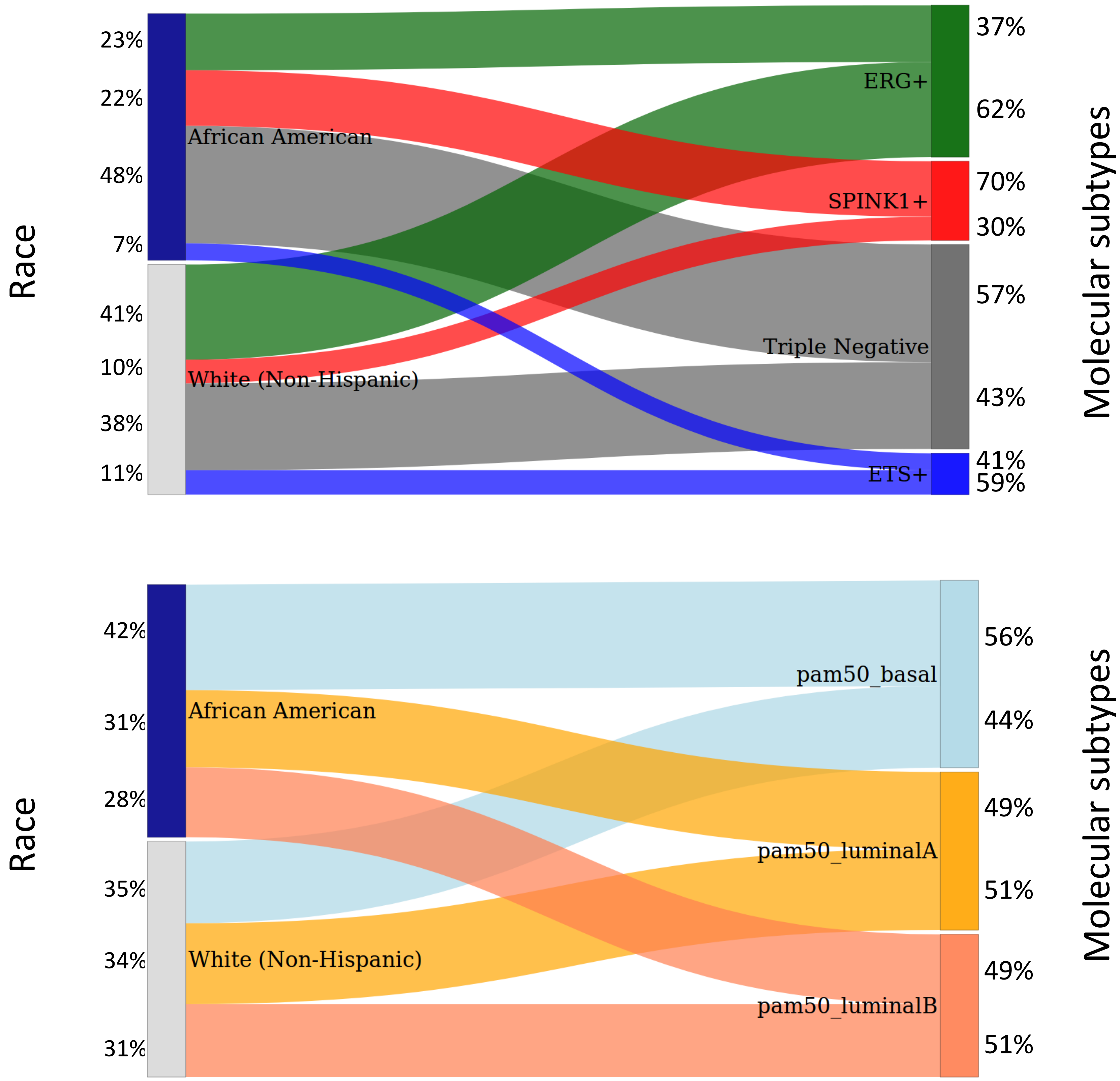

Supplemental Figure 1: Prevalence of race-based genomic subtypes in prostate cancer. Association of molecular subtypes between race and ETS-based (top) and PAM50 (bottom) subtypes. African American patients are more enriched in the SPINK1+ subtype and less enriched in the ERG+ subtype compared with White patients. However, African American patients are more enriched in basal subtypes. Patients that are ERG-, SPINK1- and ETS- are referred to as Triple negative.

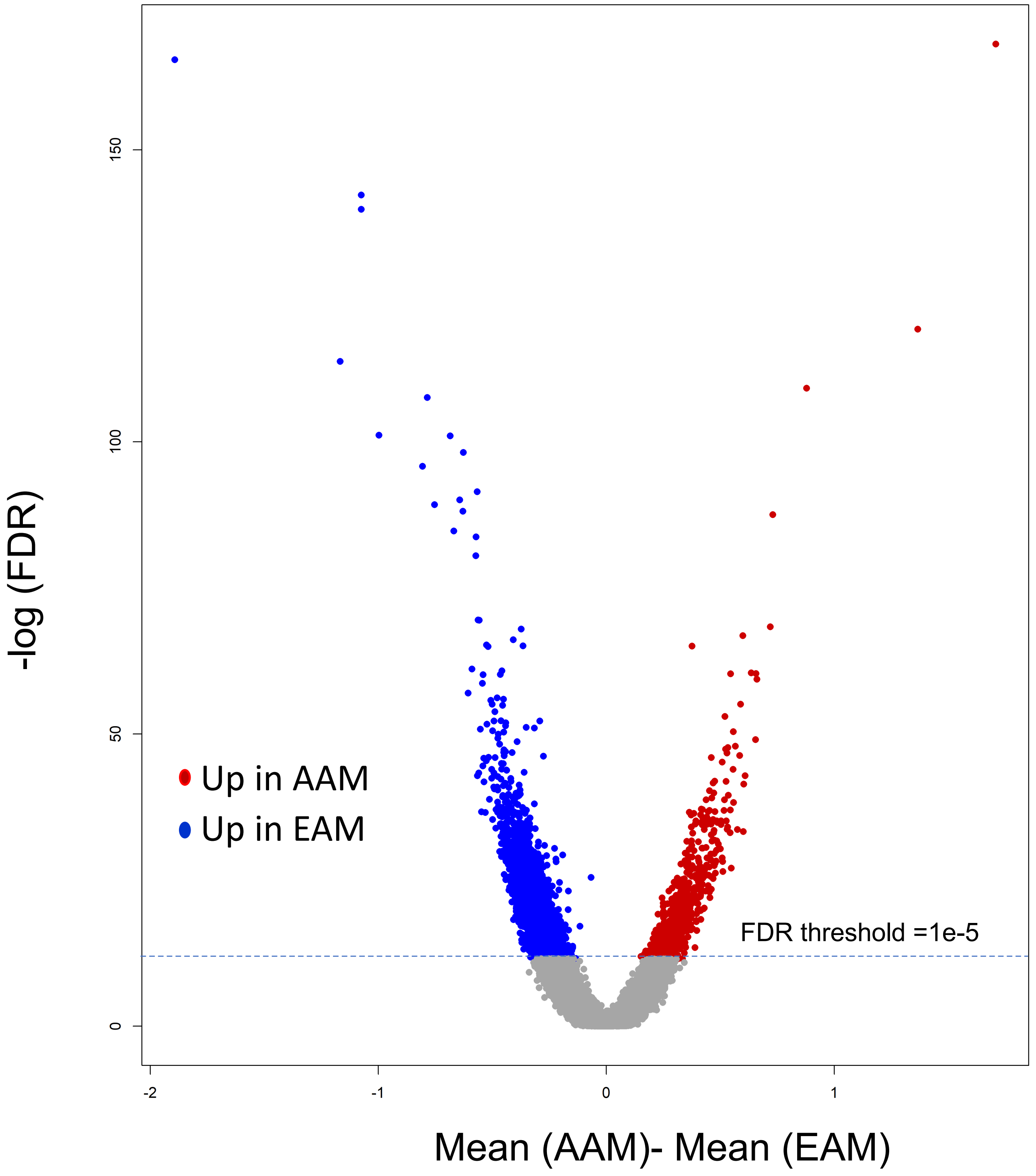

Supplemental Figure 2: Volcano plot of differentially expressed genes.
A volcano plot showing 778 genes upregulated in AAM (red) and 3807 genes upregulated in EAM (blue) in our cohort.

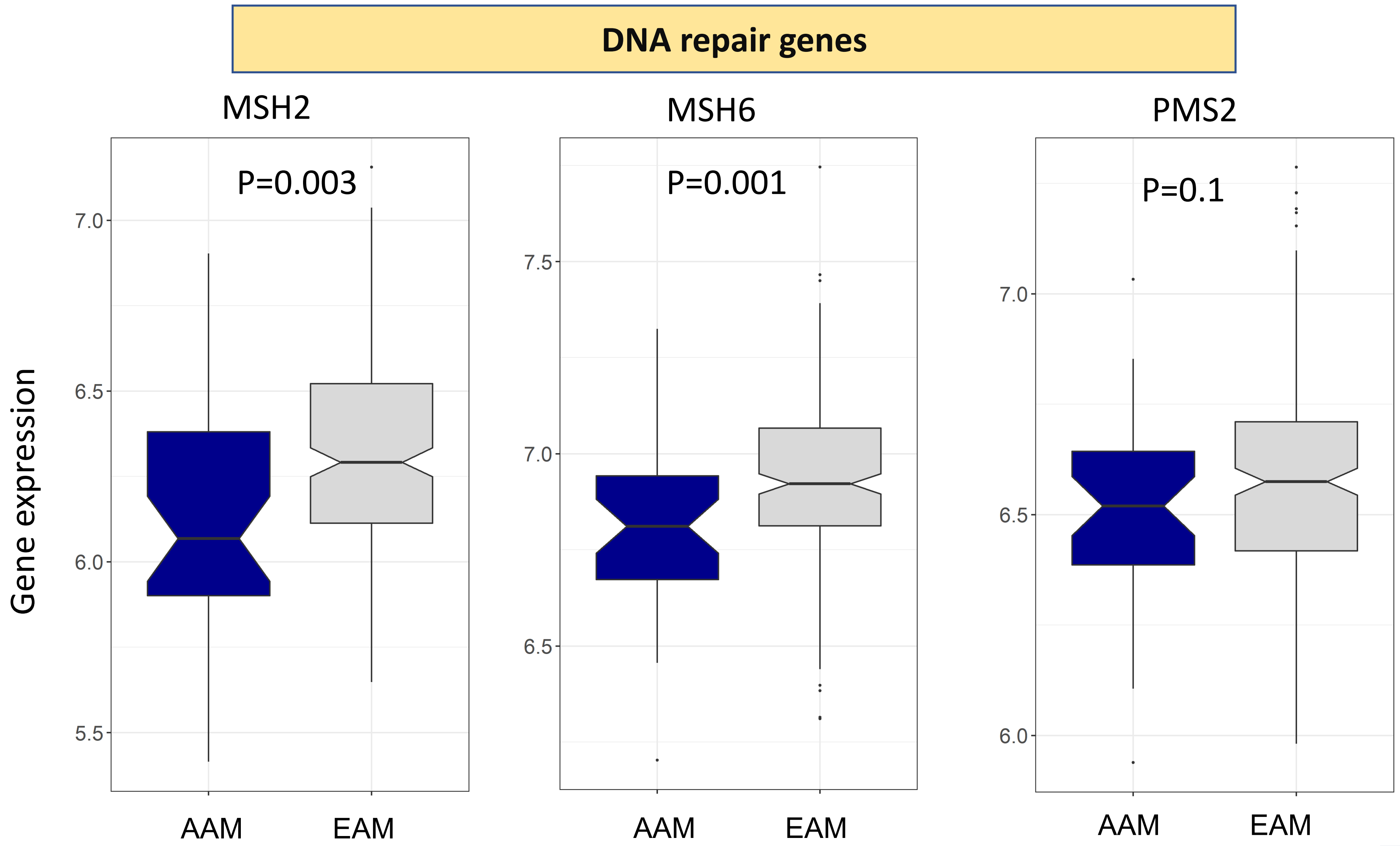

Supplemental Figure 3: Box plots of DNA repair genes, inflammation genes in TCGA cohort. Using TCGA cohort with 37 African American (AAM) and 233 European American (EAM), AAM tend to be lower in DNA repair genes and higher immune related genes. Error bars represent the 95% confidence interval.

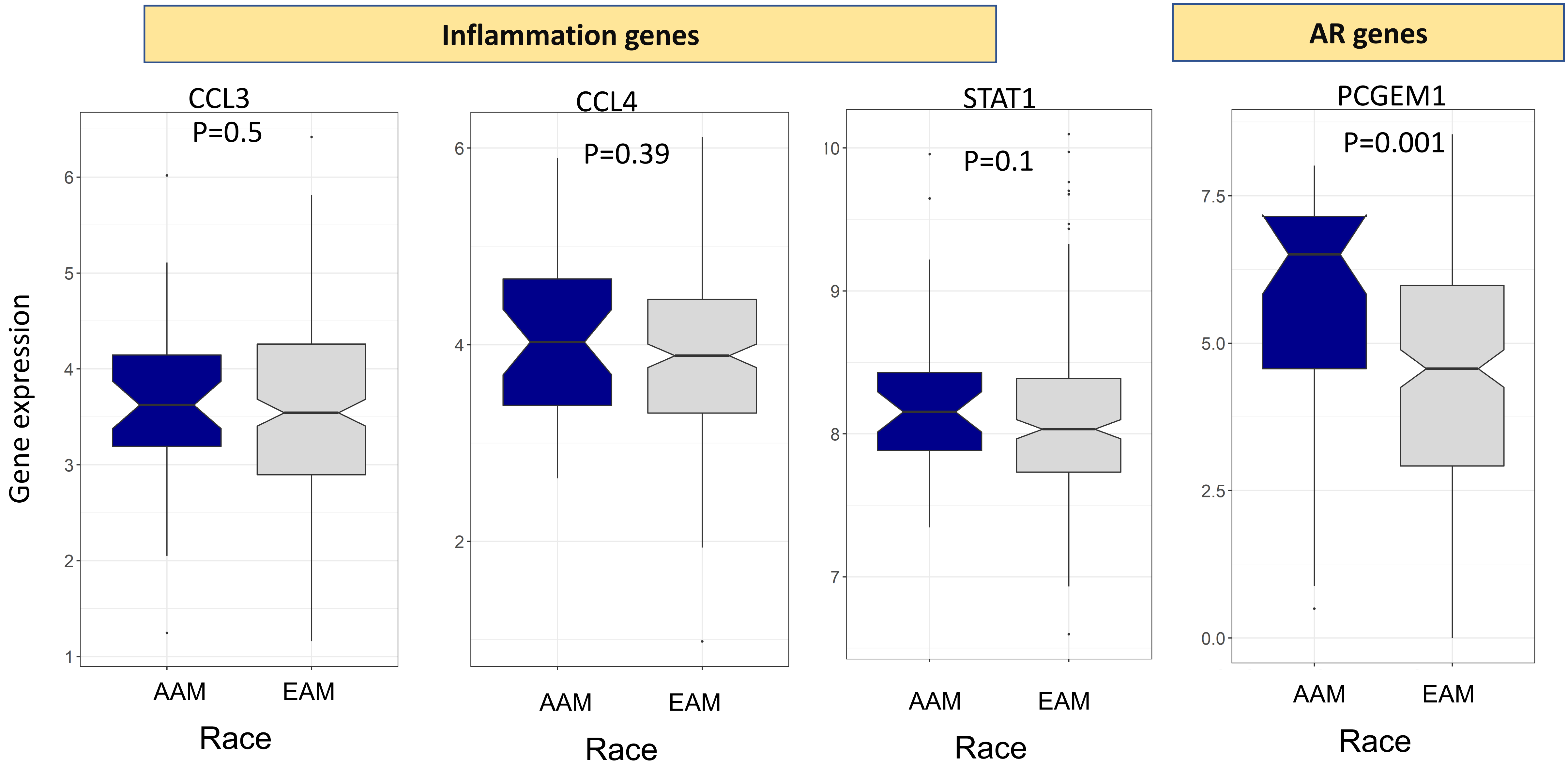

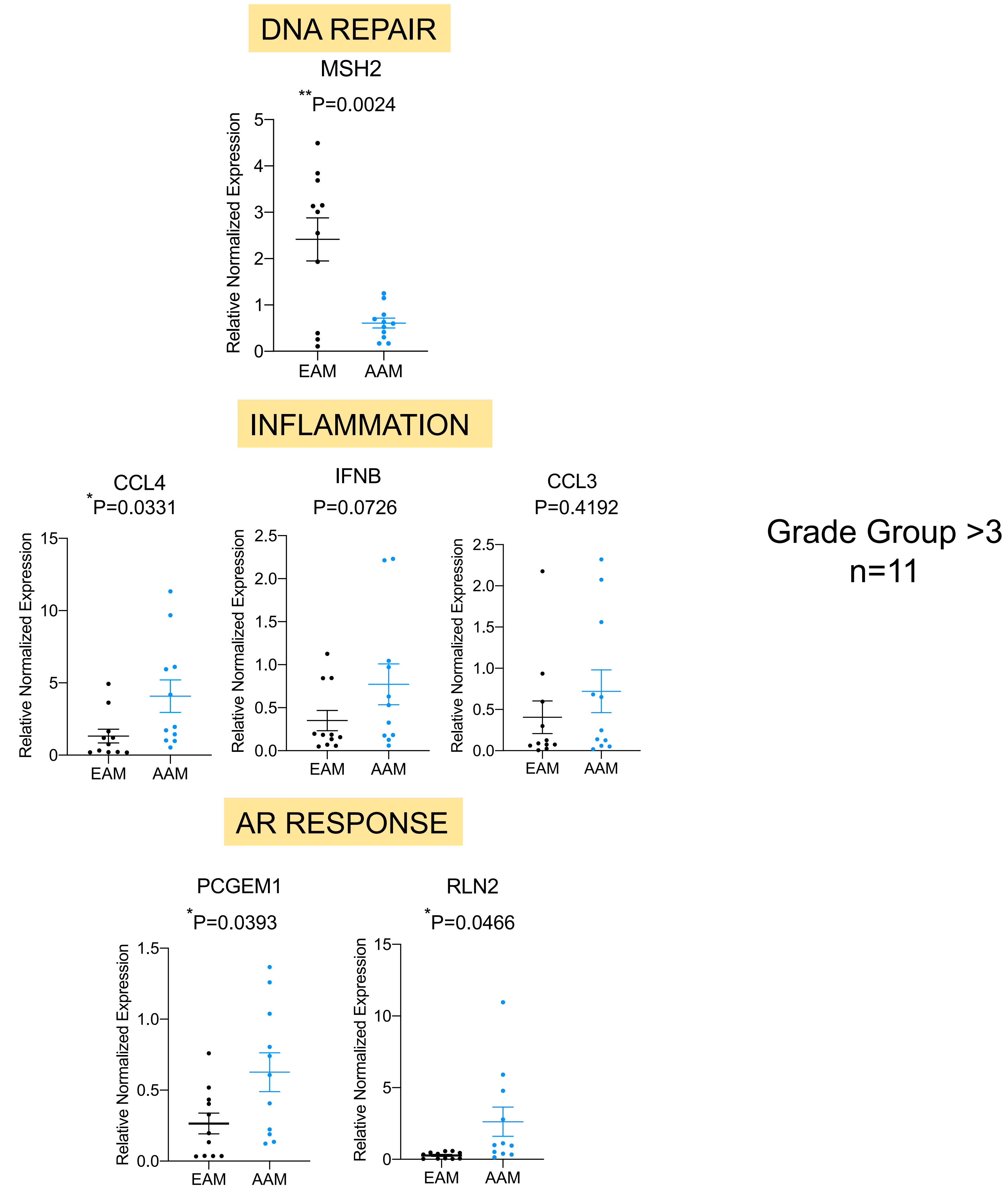

Supplemental Figure 4: External validation of DDR, immune response and AR response genes.
RNA isolated from primary prostate tissues from EAM and AAM subjects (N=11, Grade Group>3) was used in qPCR Analysis. Representative genes from DNA repair pathway (MSH2), Inflammation (CCL3, CCL4, IFNB,) and AR response (RLN2, PCGEM1) are shown. Gene expression trends confirm that immune and AR response genes are upregulated in AAM subjects while DNA mismatch repair gene MSH2 is downregulated in AAM. Unpaired t-tests was performed for comparisons (relative target gene expression) Data and Error bars are presented as mean ± SEM and *P<0.05, **P<0.005 were considered statistically significant. AAM = African American; EAM = European American. Error bars represent the 95% confidence interval.

Supplemental Table 1. Race-based chemotherapy drug-response profiling. The drug-response profile shows that African-American patients have a higher potential response rate to alkylating agent–based chemotherapy, whereas European-American patients have a higher response rate to taxane-based chemotherapy.

**Drug Name**	**Target of Inhibition**	**Agent Class**	**MVA Odds Ratio**	**MVA *P* Value**	**FDR Adjusted MVA *P* Value**
Carboplatin	DNA	Alkylating	1.09	1.25E-06	5.55E-05
Cisplatin	DNA	Alkylating	1.07	2.12E-04	9.76E-04
Methotrexate	DHFR	Anti-folate	0.91	2.70E-05	3.44E-04
Docetaxel	Tubulin	Anti-microtubule	0.94	2.05E-05	3.05E-04
Vinorelbine	Tubulin	Anti-microtubule	0.97	5.45E-04	1.73E-03
Ixabepilone	Tubulin	Anti-microtubule	0.96	6.67E-04	1.98E-03
Paclitaxel	Tubulin	Anti-microtubule	0.97	9.78E-04	2.72E-03

DHFR = dihydrofolate reductase; FDR = false discovery rate; MVA = multivariable analysis.
