# Supplementary material for: Comparative analysis of 1152 African-American and European-American men with prostate cancer identifies distinct genomic and immunological differences
Source: Commun Biol. 2021 Jun 3;4:670. doi: 10.1038/s42003-021-02140-y (PMC8175556; doi:10.1038/s42003-021-02140-y)
Supplement: Supplementary file 3 — Descriptions of Additional Supplementary Files [file 42003_2021_2140_MOESM3_ESM.pdf]

Descriptions of Additional Supplementary Files

Supplemental Data 1
Description: Cancer hallmarks pathways from MSIGDB and genes involved in pathways.

Supplemental Data 2
Description: Differential expression genes between AAM and EAM in TCGA-prostate and our cohort.

Supplemental Data 3
Description: Pathways significantly associated with AAM and EAM patients after adjusting for clinical variables and false discovery. Many of the pathways more active in AAM men are related to the immune response, whereas many of the pathways more active in EAM men are related to DNA repair, glycolytic metabolism, and the cell cycle. Pathway association with race after adjusting for clinical variables.

AAM = African American; EAM = European American; FDR = false discovery rate

Supplemental Data 4
Description: Source data for figures.

Supplemental Data 5
Description: R code used to generate figures.
