# Supplementary material for: Comparative analysis of 1152 African-American and European-American men with prostate cancer identifies distinct genomic and immunological differences
Source: Commun Biol. 2021 Jun 3;4:670. doi: 10.1038/s42003-021-02140-y (PMC8175556; doi:10.1038/s42003-021-02140-y)
Supplement: Supplementary file 9 — Reporting Summary [file 42003_2021_2140_MOESM9_ESM.pdf]

nature research

Corresponding author(s): Ash Tewari, Alp Tuna Beksac

Last updated by author(s): Oct 10, 2020

Reporting Summary

Nature Research wishes to improve the reproducibility of the work that we publish. This form provides structure for consistency and transparency in reporting. For further information on Nature Research policies, see our Editorial Policies and the Editorial Policy Checklist.

Statistics

For all statistical analyses, confirm that the following items are present in the figure legend, table legend, main text, or Methods section.

n/a	Confirmed	
☐	☒	The exact sample size (n) for each experimental group/condition, given as a discrete number and unit of measurement
☐	☒	A statement on whether measurements were taken from distinct samples or whether the same sample was measured repeatedly
☐	☒	The statistical test(s) used AND whether they are one- or two-sided<br>*Only common tests should be described solely by name; describe more complex techniques in the Methods section.*
☐	☒	A description of all covariates tested
☐	☒	A description of any assumptions or corrections, such as tests of normality and adjustment for multiple comparisons
☐	☒	A full description of the statistical parameters including central tendency (e.g. means) or other basic estimates (e.g. regression coefficient) AND variation (e.g. standard deviation) or associated estimates of uncertainty (e.g. confidence intervals)
☐	☒	For null hypothesis testing, the test statistic (e.g. F, t, r) with confidence intervals, effect sizes, degrees of freedom and P value noted<br>*Give P values as exact values whenever suitable.*
☒	☐	For Bayesian analysis, information on the choice of priors and Markov chain Monte Carlo settings
☐	☒	For hierarchical and complex designs, identification of the appropriate level for tests and full reporting of outcomes
☐	☒	Estimates of effect sizes (e.g. Cohen's d, Pearson's r), indicating how they were calculated

Our web collection on statistics for biologists contains articles on many of the points above.

Software and code

Policy information about availability of computer code

Data collection	No commercial or open source data is used. Codes are written by the authors on the study.
Data analysis	Data analysis is performed by Mohammed Alshalalfa and he used his custom codes. Statistical analysis is explained in the manuscript. Codes are available upon request.

For manuscripts utilizing custom algorithms or software that are central to the research but not yet described in published literature, software must be made available to editors and reviewers. We strongly encourage code deposition in a community repository (e.g. GitHub). See the Nature Research guidelines for submitting code & software for further information.

Data

Policy information about availability of data

All manuscripts must include a data availability statement. This statement should provide the following information, where applicable:

- Accession codes, unique identifiers, or web links for publicly available datasets
- A list of figures that have associated raw data
- A description of any restrictions on data availability

The MIAME compliant, microarray data that support the findings of this study were deposited on NCBI Gene expression Omnibus (GEO) and are accessible through GEO accession number GSE169038. Additional data used in this study is available upon request from the PI of this study (Dr. Ash Tewari).

Field-specific reporting

Please select the one below that is the best fit for your research. If you are not sure, read the appropriate sections before making your selection.

☒ Life sciences ☐ Behavioural & social sciences ☐ Ecological, evolutionary & environmental sciences

For a reference copy of the document with all sections, see nature.com/documents/nr-reporting-summary-flat.pdf

Life sciences study design

All studies must disclose on these points even when the disclosure is negative.

Sample size	Data of all available patients have been used for analysis. A power analysis is not made. Despite the lack of power analysis the sample should be considered sufficient as this study has the highest number of African American patients with prostate cancer in literature.
Data exclusions	This data only includes patients who had the commercially available Decipher test performed after radical prostatectomy. Any patient without the test were excluded due to lack of sequencing data.
Replication	The methodology is thoroughly explained and is easily reproducible. We have performed similar analyses with the commercially available TCGA dataset and a small sample size from a different clinical trial.
Randomization	Patients were divided into two groups based on race.
Blinding	not applicable to our study

Reporting for specific materials, systems and methods

We require information from authors about some types of materials, experimental systems and methods used in many studies. Here, indicate whether each material, system or method listed is relevant to your study. If you are not sure if a list item applies to your research, read the appropriate section before selecting a response.

Materials & experimental systems

n/a	Involved in the study	
☒	☐	Antibodies
☒	☐	Eukaryotic cell lines
☒	☐	Palaeontology and archaeology
☒	☐	Animals and other organisms
☐	☒	Human research participants
☐	☒	Clinical data
☒	☐	Dual use research of concern

Methods

n/a	Involved in the study	
☒	☐	ChIP-seq
☒	☐	Flow cytometry
☒	☐	MRI-based neuroimaging

Human research participants

Policy information about studies involving human research participants

Population characteristics	All patients who underwent radical prostatectomy for localized or locally advanced prostate cancer and subsequently underwent a commercially available genomic testing done.
Recruitment	All patients included in the study underwent radical prostatectomy for prostate cancer.
Ethics oversight	Institutional Review Board at Icahn School of Medicine at Mount Sinai approved the study.

Note that full information on the approval of the study protocol must also be provided in the manuscript.

Clinical data

Policy information about clinical studies

All manuscripts should comply with the ICMJEguidelines for publication of clinical research and a completedCONSORT checklist must be included with all submissions.

Clinical trial registration	n/a
Study protocol	IRB protocol information can be requested from the PI Dr. Ash Tewari.
Data collection	The clinical data used in the study is submitted to Decipher Biosciences prior to the Decipher testing. Any missing information is gathered by authors from the Icahn School of Medicine at Mount Sinai

Outcomes	Study outcomes were designed according to the previously published manuscripts regarding the molecular alterations that have association with prostate cancer.
